# Status of the application of artificial intelligence (AI) in robotic surgical skills training in the modern robotic era and future horizons: a systematic review

**DOI:** 10.1007/s00464-026-13396-y

**Published:** 2026-09-28

**Authors:** Shuntong Zhang, Changyou Tang, Pradeep Patil, Benjie Tang

**Affiliations:** 1 Division of Respiratory Medicine and Gastroenterology, Dundee Institute for Healthcare Simulation, School of Medicine, University of Dundee, Dundee, UK; 2 Edinburgh Medical School, University of Edinburgh, Edinburgh, UK; 3 Department of General Surgery, Ninewells Hospital and Medical School, NHS Tayside, University of Dundee, Dundee, UK

**Keywords:** Robotic surgical skills training, Artificial intelligence (AI), Machine learning (ML), AI-based feedback, Da Vinci simulator, Technical robotic skills assessment

## Abstract

**Background:**

Artificial intelligence (AI) is a rapidly evolving field with increasing applications in surgical education and assessment; however, its integration into robotic surgical skills training remains in the early stages. The aim of this systematic review is to evaluate the current state of AI applications in robotic surgical skills training and outline future research directions for integrating AI into robotic surgical skills training.

**Method:**

This review was reported in accordance with the PRISMA 2020 statement. A systematic search was performed across PubMed, Scopus, Web of Science, the Cochrane Library, and Ovid Embase. Studies were eligible for inclusion if they applied artificial intelligence (AI) methods to robotic surgical skills training, were published in English, and were published between 1 January 2000 and 1 October 2025. Risk of bias was assessed using the Cochrane RoB 2 tool and the ROBINS-I tool.

**Result:**

Of the 4400 studies initially identified, 36 met the inclusion criteria and were included in the final analysis. Among these, 12 were assessed as having a low risk of bias, while 24 were judged to present some concerns; studies with a high risk of bias were excluded. All included studies employed AI-based platforms, with the Da Vinci Skills Simulator (dVSS) being the most frequently used system. AI methods were applied to support surgical skills development, with Suturing and Needle Passing emerging as the most evaluated tasks. AI-based feedback, AI training methodologies and models, and AI-based assessment are also employed.

**Conclusion:**

AI-based simulators and training methods have been developed for robotic surgical skills training and are increasingly used to enhance the technical abilities of trainees in robot-assisted surgery (RAS). However, the integration of AI and robotic surgical skills training is still at an early stage. Lack of standardized implementation and limited integration of AI tools into formal robotic surgical skills training continue to hinder broader adoption.

**Trial registration:**

This systematic review was registered with PROSPERO, registration number is CRD420251178615.

**Graphical abstract:**

**Figure:**
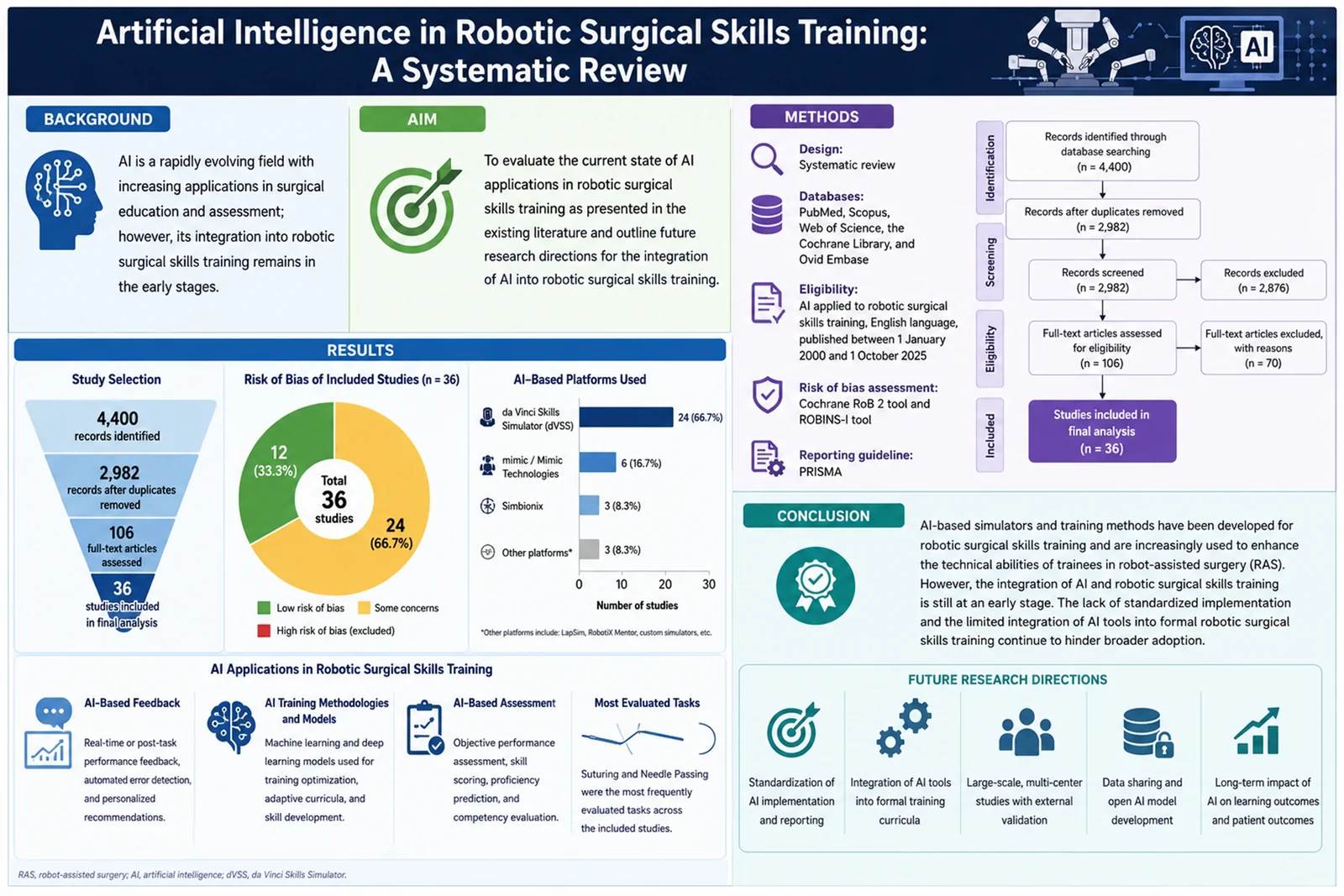

**Supplementary Information:**

The online version contains supplementary material available at https://doi.org/10.1007/s00464-026-13396-y.

The approval of the Da Vinci Surgical System by the U.S. Food and Drug Administration (FDA) in 2000 established robot-assisted surgery (RAS) as a viable and transformative surgical modality [1]. The Da Vinci Surgical System is based on a master–slave architecture and consists of three primary components: a surgeon console that enables visualization of the operative field and control of the endoscope and instruments; a patient-side cart equipped with three to four articulated robotic arms that manipulate the endoscope and surgical tools; and a set of wristed minimally invasive instruments offering up to seven degrees of freedom, complemented by a high-definition three-dimensional vision system [2]. Robotic surgery offers several potential advantages, including enhanced precision, greater instrument dexterity, three-dimensional visualization, and improved surgeon ergonomics, which may facilitate complex procedures. However, it also has limitations, including high acquisition and maintenance costs, longer setup times, and the absence of tactile feedback [3–5].

Due to the complexity of RAS, the learning curve for this technique is steep and lengthy, and there is no unified criterion to determine whether a surgeon has reached proficiency. In RAS, one surgeon typically operates the console at any given time, however, a dual console system is available setting, which allows two surgeons to operate collaboratively when necessary, particularly for/during training and mentorship purposes \* MERGEFORMAT [6]. Unlike conventional open surgery, RAS presents surgeons with a magnified video feed for visualization, necessitating adaptation to processing information from this perspective [7]. Furthermore, the procedure demands complex eye-hand, bimanual, and active foot coordination [8], which collectively contributes to a significantly higher mental workload for the surgeon. During the training phase, novice surgeons may pose potential risks to patient safety due to operational errors. Therefore, effective and safe robotic surgical skills training is of critical importance.

Simulation-based training has emerged as an essential approach for developing robotic surgical skills. Traditional training modalities—including box trainers, animal or cadaveric models, hybrid simulators, and virtual reality (VR) platform have inherent limitations, such as excessive cost, limited tissue fidelity, and a lack of objective performance assessment [9]. In recent years, artificial intelligence (AI) has been increasingly integrated into surgical education and training, offering novel solutions to these challenges. As an AI-based training platform, the Da Vinci Simulator enables trainees to achieve technical skills proficiency in a safe environment without exposing patients to procedural risks. It provides highly realistic surgical environments that allow repeated practice, reducing the need for human supervision and intervention. The simulator continuously updates trainees’ skill sets through real-time feedback and accelerates learning curves for complex tasks. As a result, it enhances technical proficiency and better prepares trainees for real-world clinical scenarios [10]. AI-driven systems also enable personalized training pathways, objective performance evaluation, addressing many shortcomings of traditional training methods [11]. Within the surgical specialty, four major subsets of AI are most integrated into training programs: machine learning, natural language processing, artificial neural networks, and computer vision [12]. These AI methods allow for more effective and tailored training and help to reduce the initial learning curve that prepares trainees for real-world clinical challenges. Therefore, the integration of AI into robotic surgical skills training is important and represents a promising future direction in surgical education.

AI is a rapidly evolving field with increasing applications in surgical education and assessment; however, its integration into robotic surgical skills training remains in the early stages. The aim of this systematic review is to evaluate the current state of AI applications in robotic surgical skills training, critically appraise AI-based robotic surgical simulators reported in the literature, and analyze experimental tasks and assessment methods employing AI. Furthermore, this review highlights existing limitations and outlines future research directions for the integration of AI into robotic surgical skills training.

## Methodology

This systematic review was reported in accordance with the guidelines of the Preferred Reporting Items for Systematic Reviews and Meta-Analyses (PRISMA) [13]. The review protocol was designed and registered in the International Prospective Register of Systematic Reviews (PROSPERO); registration number is CRD420251178615.

### Search strategy

PubMed, Scopus, Web of Science, the Cochrane Library, and Ovid Embase were systematically searched from January 1, 2000 to October 1, 2025. The search strategy combined medical subject headings (MeSH) and terms related to artificial intelligence and robotic surgical skills training, including (“machine learning” OR “artificial intelligence” OR “AI” OR “deep learning”) AND (“Da Vinci training” OR “robotic surgical skills training” OR “Da Vinci simulator” OR “robotic surgery training” OR “technical robotic skills assessment” OR “Fundamental Skills of Robotic Surgery” OR “FSRS”). Reference lists of included studies were manually screened to identify additional relevant articles.

### Eligibility criteria

Our inclusion criteria comprised original research articles that applied AI or machine learning methods to robotic surgical skills training. Studies were excluded if they did not involve AI or machine learning, focused on non-robotic surgical platforms, or evaluated surgical training delivered through video observation or expert guidance alone. We also excluded studies that used AI solely to differentiate between expert and novice surgeons, as well as those applying AI for RAS imaging or for visual simulation outside of a training setting, tactile simulation, or mechanical simulation rather than skills training. In addition, review articles, systematic reviews, conference proceedings, comments, letters to the editor, and non-English language publications were excluded (Table 1).

**Table 1:** Inclusion and exclusion criteria

Inclusion Criteria	Exclusion criteria
English language	Non-English language
Original research articles	Review articles, systematic reviews, conference abstracts, comments, and letters to the editor
Used AI methods in robotic surgical skills training	Robotic surgical skills training without AI
	Non-robotic platforms
	Surgery training carried out by watching videos or expert guidance
	Distinguish experts and novices outside of a training setting
	Use AI in surgery outside of a training setting

### Study selection

Before the initial screening, EndNote 2025 was used to remove duplicate records retrieved from the five databases. Two authors (STZ and BJT) then independently screened the titles and abstracts using EndNote 2025. Full-text articles of studies that passed the initial screening were subsequently assessed for eligibility. Studies that did not meet the eligibility criteria after full-text review were excluded. Any disagreements between the two reviewers at either the screening or full-text assessment stage were first resolved through discussion; if consensus could not be reached, the second author (CYT) and third author (PP) acted as arbitrators and made the final decision.

### Data extraction

Data extraction was performed by two independent authors (STZ, BJT) and discussed for resolution. Data extracted included author and year, title, platform, task, evaluations, and comments. To address heterogeneity across simulator platforms, study years, and data acquisition settings, the findings of this review were synthesized using a narrative approach rather than a quantitative meta-analysis. Studies were compared at the level of task type, simulator platform, and reported evaluation metrics, allowing for structured qualitative interpretation while acknowledging differences in underlying data sampling rates and parameter definitions.

### Risk of bias

Two authors (STZ and BJT) independently assessed the risk of bias. Version 2 of the Cochrane risk of bias tool (RoB 2) was used for randomized controlled trials, and the Risk of Bias in Non-randomized Studies of Interventions (ROBINS-I) tool was used for non-randomized controlled trials. In the event of a disagreement, the second author (CYT) and third author (PP) acted as arbitrators and made the final decision.

## Results

**Fig. 1:**
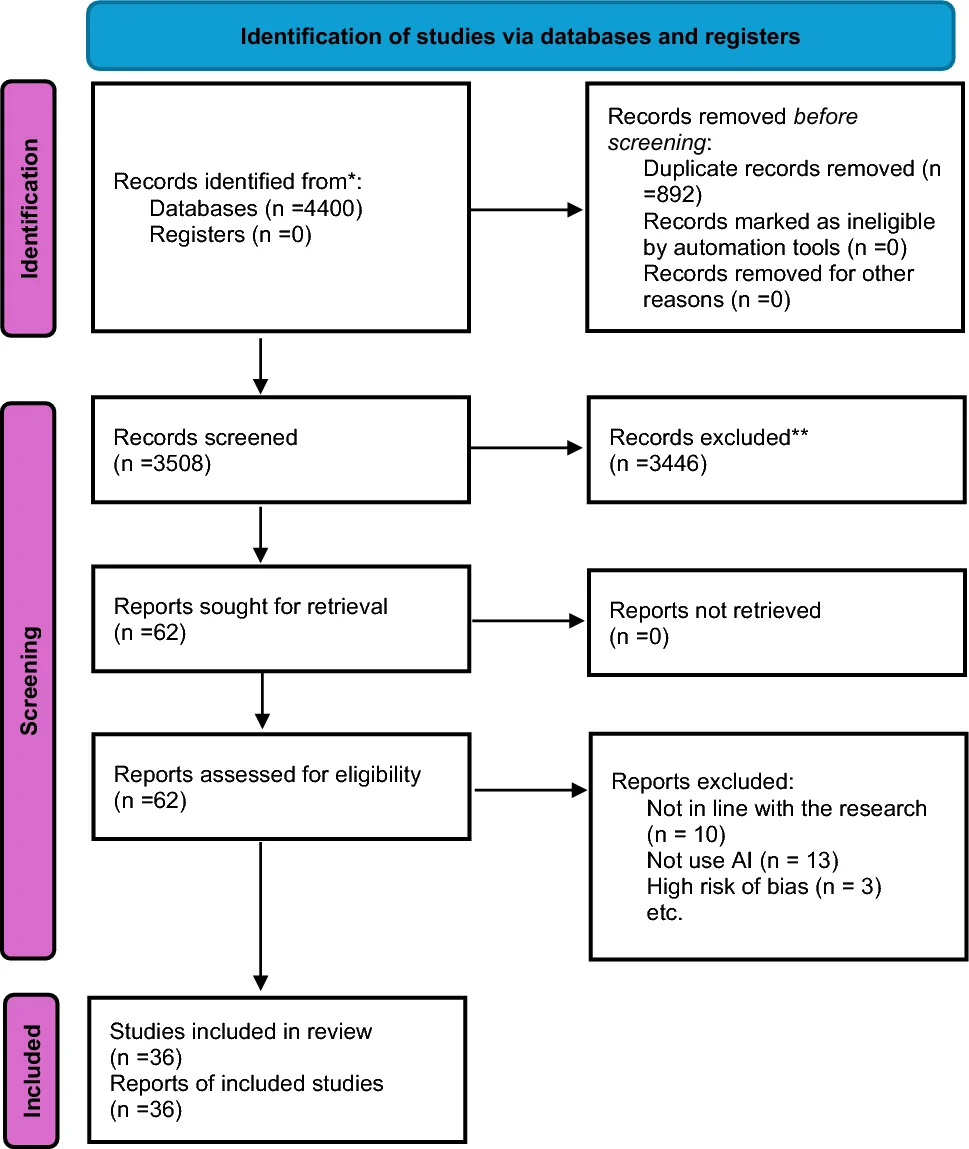
Literature identification process following the preferred reporting items for systematic reviews and meta-analyses (PRISMA) guidelines

A systematic search of the databases yielded a total of 4400 records. After the removal of 892 duplicate entries, 3508 unique records remained for initial screening. The titles and abstracts of these records were then assessed, leading to the exclusion of 3446 irrelevant publications. Subsequently, the full texts of the remaining 62 reviews were retrieved and evaluated against the inclusion criteria. Following this full-text review, 26 papers were excluded, resulting in a final selection of 36 eligible reviews that were included in the analysis (Fig. 1).

### Risk of bias

**Fig. 2:**
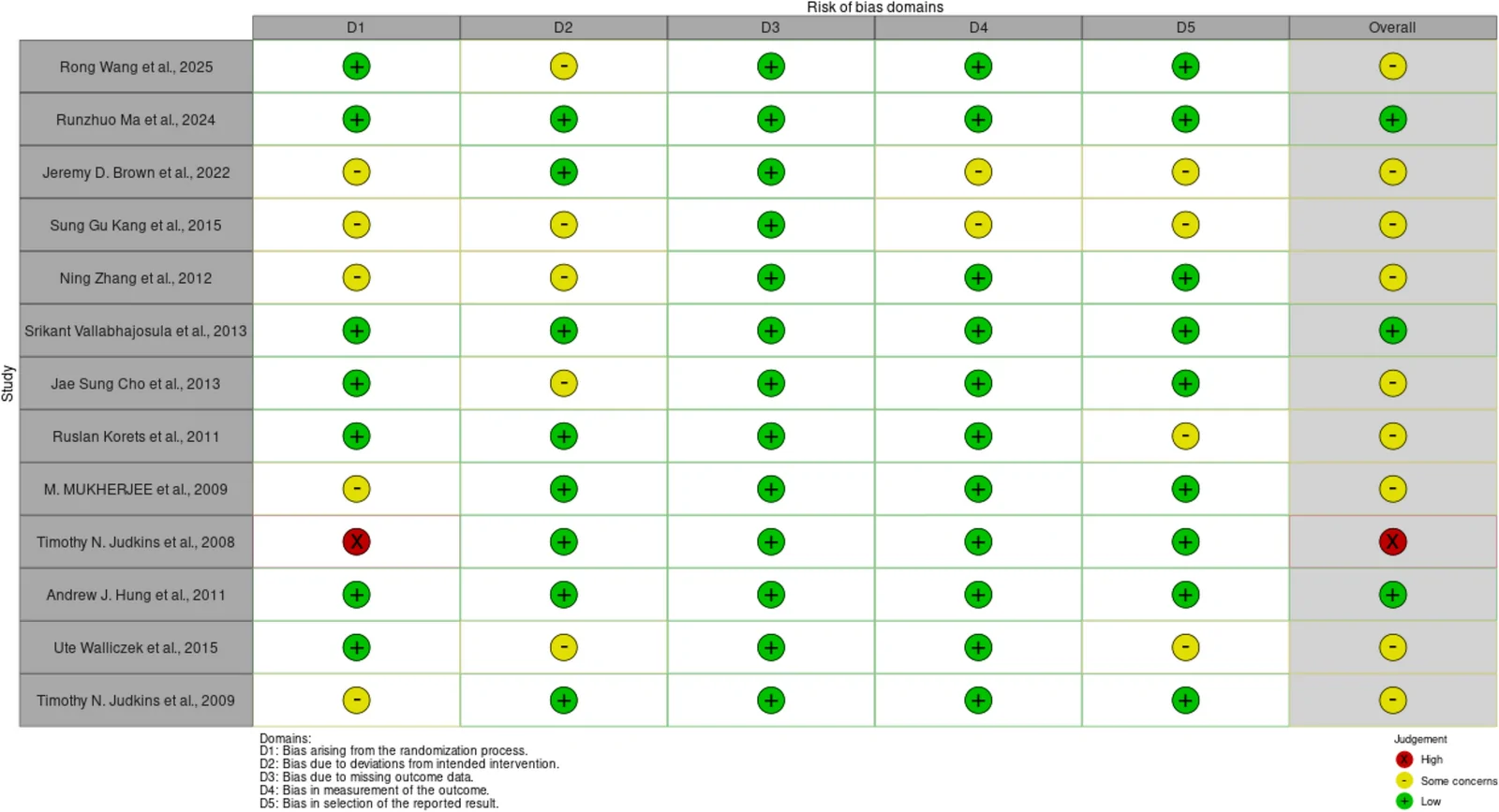
Version 2 of the Cochrane risk of bias tool (RoB 2) in randomized controlled trials studies

**Fig. 3:**
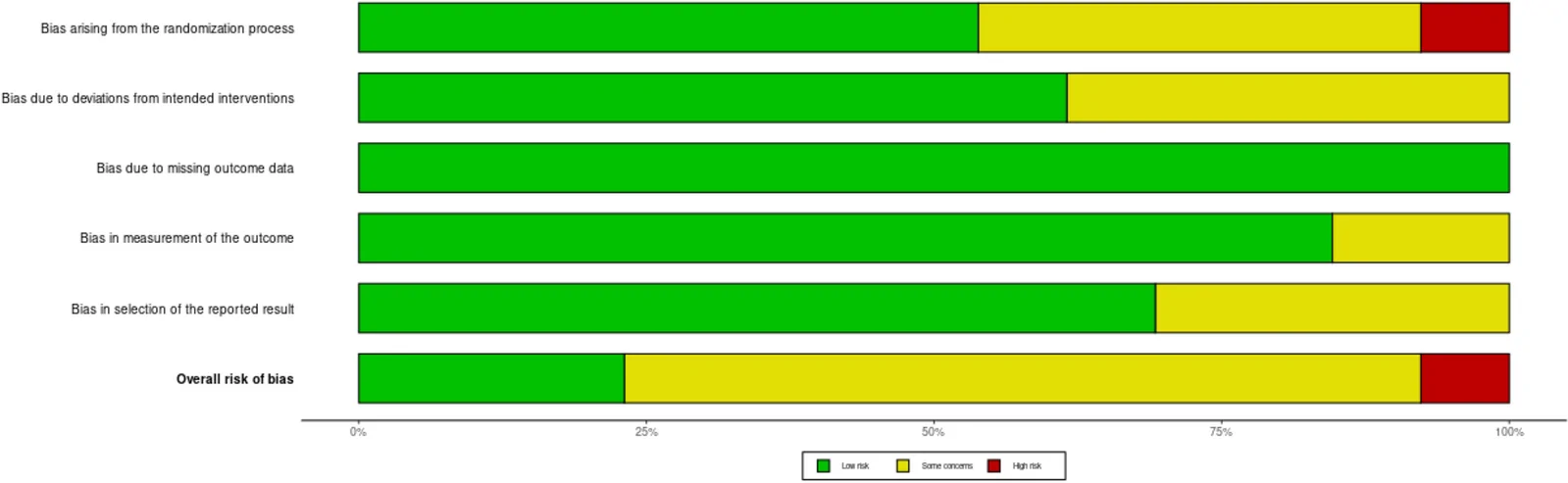
Summary of Version 2 of the Cochrane risk of bias tool (RoB 2) across bias

**Fig. 4:**
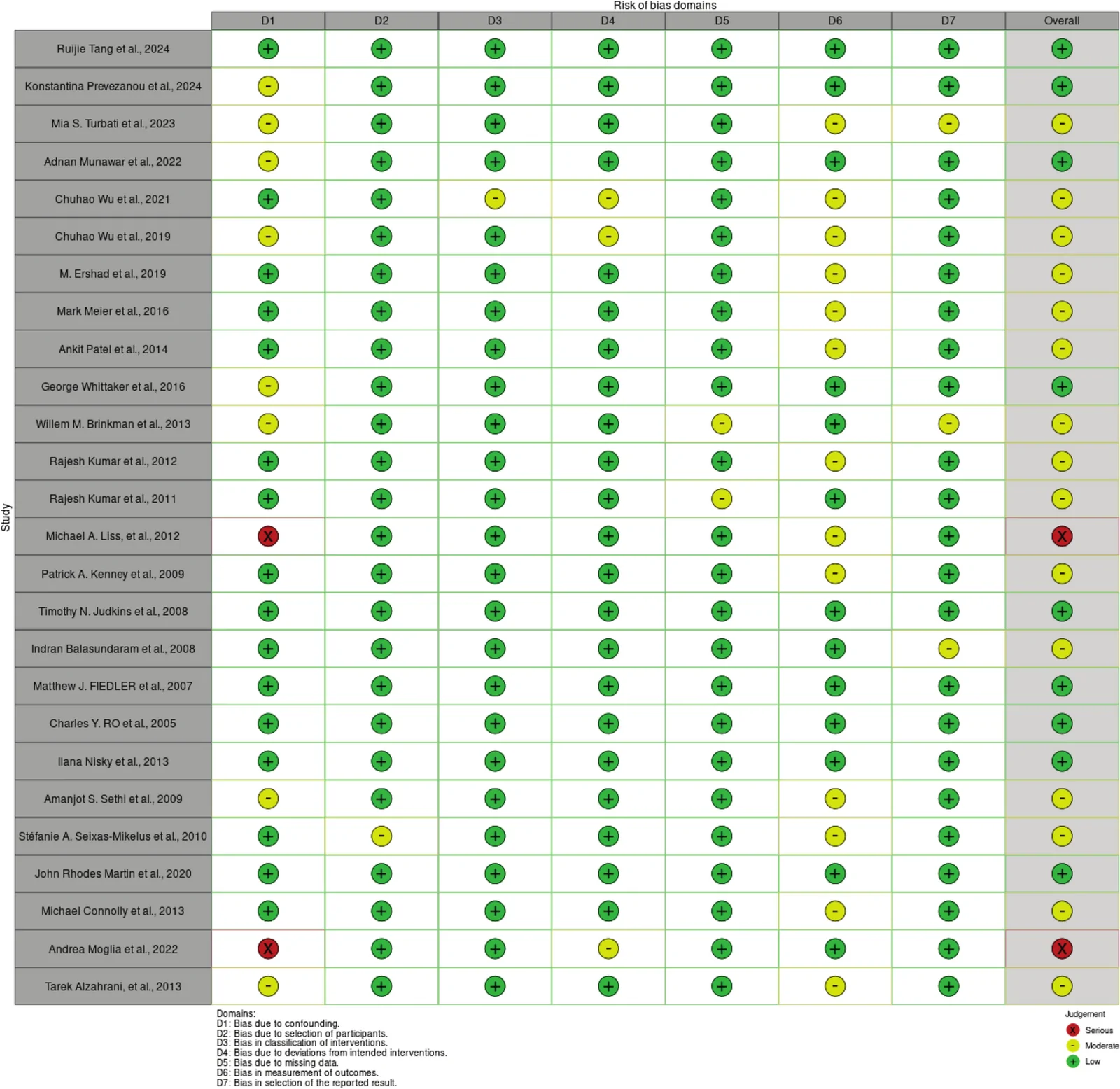
Risk of bias in non-randomized studies of interventions (ROBINS-I) in non-randomized controlled trials studies

**Fig. 5:**
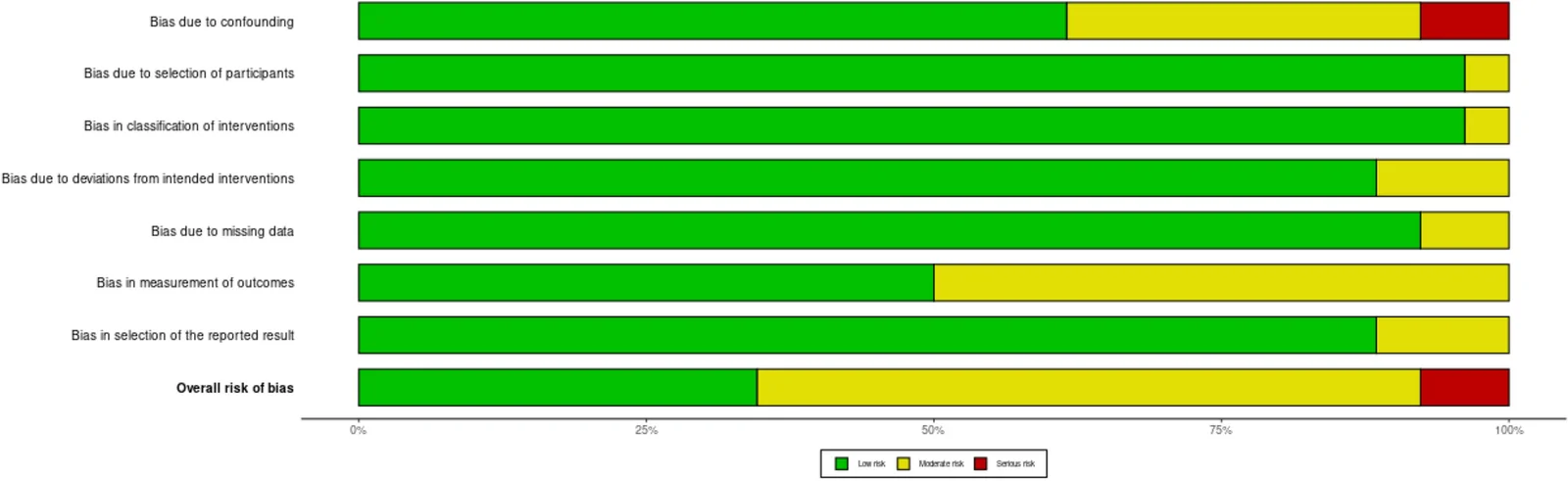
Summary of Risk of Bias In Non-randomized Studies of Interventions (ROBINS-I) across bias domains

The results of the risk of bias assessment are shown in Figs. 2, 3, 4, and 5. Among all the 39 articles that underwent the assessment of bias risk, twelve studies were assessed to have low risk of bias, twenty-four studies were assessed to have some concerns risk of bias, and three studies were assessed to have high risk of bias. To eliminate the influence caused by the higher risk of bias, three articles with high risk of bias was excluded.

### Characteristics of included studies

The characteristics of the 36 studies included in this systematic review are summarized in Table 2. It comprises 12 randomized controlled trials [14, 17, 19, 25, 28, 29, 31, 34, 35, 44, 47, 49] and 24 non-randomized controlled trials [15, 16, 18, 20–24, 26, 27, 30, 32, 33, 36–43, 45, 46, 48]. The publication dates of the studies included in this review range from 2005 to 2025. The sample size of all the studies ranged from 7 participants [21] to 48 participants [48], with an average of 22 participants. Of the studies included in the review, all the platforms are based on AI. Among them, 27 are Da Vinci Skills Simulator (dVSS), 1 is LAP MentorTM laparoscopic simulator [16], 3 are Mimic dV-Trainer (MdVT) [25, 33, 40], 2 are RobotiX Mentor [27, 46], and 3 are self-developed platforms [15, 20, 23].

**Table 2:** Summary of 36 included studies

Author, year	Platform	AI method			Task		Evaluations	Comments
Rong Wang et al., 2025 [14]	Da Vinci Skills Simulator	AI in Simulators			Each participant completed six tasks on three separate occasions while fitted with electroencephalography (EEG) equipment		Relative to the control group, the VR-training group exhibited significantly superior GEARS scores (24.91 ± 3.36 vs. 19.68 ± 3.07; *p* < 0.01) and dVSS scores, alongside a significantly lower NASA TLX score (40.04 ± 10.55 vs. 48.2 ± 9.88; *p* < 0.01)	Training on the dVSS simulator significantly enhanced fundamental robotic surgical proficiency while simultaneously reducing operator workload
Ruijie Tang et al., 2024 [15]	Self-designed needle insertion simulators	LS, FNN			Introduces and evaluates two data-driven methods for estimation: least-square (LS) regression and feedforward neural networks (FNNs)		The FNN achieved over 90% accuracy, outperforming the LS model, which attained a mean accuracy of 78.02% across the defined evaluation metrics	The FNN models significantly outperformed the LS model across all devised evaluation metrics
Konstantina Prevezanou et al., 2024 [16]	LAP MentorTM laparoscopic simulator	KNN, LDA, GNB, QDA, LR, RF, SVM, deep learning			A virtual reality training course consisting of nine different tasks		The results demonstrated that for two-class classification, the model achieved accuracies of 97.1%, 96.9%, and 75.7% for Task 5, 6, and 7, respectively. In three-class classification, the corresponding accuracies were 96.3%, 95.9%, and 99.7%	The created models hold promises for direct application in real-life training settings, offering a scalable solution for the automated, trainee-specific assessment of surgical skills
Runzhuo Ma et al., 2024 [17]	Da Vinci Skills Simulator	AI in Simulators			Completing two rounds of suturing tasks		When observing skill improvement from R1 to R2, the feedback group demonstrated a significantly greater improvement in needle handling score compared to the control group (0.30 vs − 0.02, *p* = 0.018)	AI-based feedback facilitates the acquisition of robotic surgical skills among trainees, especially underperformers
Mia S. Turbati et al., 2023 [18]	The SimNow resident robotic basic simulation curriculum	AI in Simulators			Each participant performed three scored trials		On average, competent and expert surgeons achieved significantly higher overall scores (90.4–92.8%) compared to novices (70.5%; *p* = 0.02 and *p* = 0.01, respectively). Furthermore, experts reported a substantially lower perceived workload than novices (15.8 vs. 45.8; *p* = 0.02), indicating greater ease during task performance	The SimNow curriculum represents an effective component of a comprehensive robotic surgical curriculum for general surgery residents
Jeremy D. Brown et al., 2022 [19]	Da Vinci Skills Simulator	Machine learning			Performed five trials of inanimate peg transfer		Participants gave an average rating of 93 ± 14 (0–100 scale) for the control group and gave an average rating of 86 ± 12 (0–100 scale) for the feedback group	While automated skill evaluation systems may enhance trainees’ self-awareness, they do not appear to expedite the acquisition of early-stage psychomotor skills in robotic surgical training
Adnan Munawar et al., 2022 [20]	A self-designed standardized simulation platform	Machine learning			An example of the suturing procedure on a phantom model is shown, which has been established as an open benchmark challenge for the research community		The principal contribution of this work lies in the open-source release of a dynamic simulation environment for realistic phantom suturing	These features create a flexible platform for simulation-based surgical training and foundational research in machine learning for surgical scene understanding and autonomous execution
Chuhao Wu et al., 2021 [21]	Da Vinci Skills Simulator	Machine learning classification			The tasks targeted core skills such as camera control, EndoWrist manipulation, clutching, needle control, and needle driving		Performance variations were linked to shifts in engagement index (rrm = 0.25, *p* < 0.001) and gaze entropy (rrm = 0.37, *p* < 0.001). Furthermore, changes in cognitive and behavioral states predicted training outcomes with 72.5% accuracy	Cognitive and behavioral metrics were found to be correlated with inter-session performance changes
Chuhao Wu et al., 2019 [22]	Da Vinci Skills Simulator	Machine learning classifiers, NB			Objects were transferred or sutured using camera control, EndoWrist manipulation, clutching, needle control, and needle driving		A positive correlation (r = 0.51) was observed between gaze entropy and perceived workload. Moreover, a classification model based on eye-tracking features predicted discrete workload levels with 84.7% accuracy	Eye-tracking measures can identify perceived workload during robotic tasks. They can not only identify specific task elements that contribute to high cognitive load but also serve as objective metrics for process-oriented assessment in robotic surgical training
M. Ershad et al., 2019 [23]	A sparse coding framework	SVM			Fourteen subjects performed two tasks on a Da Vinci Skills Simulator. Each task was repeated three times		Relative to raw data, this approach achieved a mean accuracy improvement of 18.7 to 68.5%; compared to PCA features, the gain ranged from 1.35 to 20.59%	The ability to automatically assess a trainee’s movement patterns in short time intervals would enable the delivery of real-time personalized feedback, thereby enhancing performance during surgical tasks
Mark Meier et al., 2016 [24]	Da Vinci Skills Simulator	AI in Simulators			Each participant performed three repetitions of Camera Targeting 2, Ring Walk 1, and Peg Board 2		Experienced robotic surgeons reached and maintained expert-level performance from the first three repetitions onward, all other groups performed worse	The da Vinci Skills Simulator provides an effective, accessible platform for all surgeons learning or performing RAS. Early identification of surgical talent is neither feasible nor required
Sung Gu Kang et al., 2015 [25]	Mimic dV-Trainer	AI in Simulators			Participants completed Tube 2 independently, without proctoring		The 1 h-daily practice group completed Tube 2 significantly faster than the 1 h-weekly group (*p* < 0.011)	Four consecutive days of daily one-hour practice proved most effective, yielding superior final scores and continuous improvement while significantly reducing fatigue compared to other regimens
Ankit Patel et al., 2014 [26]	Da Vinci Skills Simulator	Mixed linear models			Data were collected from the Camera Targeting, Energy Dissection, and Energy Switching exercises		According to the models, most users require an average of four to five attempts to reach 80% proficiency on any given metric	General surgeons can enhance their robotic surgical skills using virtual reality simulators like the dv-Trainer
George Whittaker et al., 2016 [27]	RobotiX Mentor	AI in Simulators			Each participant performed nine surgical tasks distributed across two modules		Experts performed better than intermediates about time taken to complete the first (*p* = 0.002) and second (*p* = 0.043) module	The RobotiX Mentor demonstrated established construct, face, and content validity. Additionally, its feasibility and acceptability for integration into surgical training curricula were confirmed
Ning Zhang et al., 2012 [28]	Da Vinci Skills Simulator	AI in Simulators			The 12 selected exercises were deemed relevant to TORS		Final training scores for all participants, the STT group, and the LTT group demonstrated significant improvement from initial to final training scores (*P* = < 0.001, 0.002, and < 0.001, respectively)	Physicians in training can acquire competency in RAS. While participants who acquired skills more rapidly also regained them faster after a training hiatus, all participants ultimately achieved equivalent competence upon retraining
Srikant Vallabhajosula et al., 2013 [29]	Da Vinci Skills Simulator	AI in Simulators				Training in 3 tasks: simple task, bimanual carrying (BC); intermediate task, needle passing (NP); and complex task, suture tying (ST)	Significant interaction for TTC and curvature showed that RP feedback training led to a greater performance improvement on the complex ST task relative to the simple BC task	Augmented feedback tailored to specific tasks enhances RAS skill acquisition. In particular, RP and grip force feedback may benefit from training for complex procedures
Willem M. Brinkman et al., 2013 [30]	Da Vinci Skills Simulator	AI in Simulators				Performed 5 repetitions of the “ring and rail II” task	Significant learning gains were observed within 1–10 repetitions across five parameters: overall score, completion time, instrument collision, instruments out of view, and critical errors (all *P* < 0.05)	While most parameters suggest that basic robotic skills can be acquired rapidly on the Da Vinci Skills Simulator, ten repetitions proved insufficient for most novices to reach expert-level performance
Jae Sung Cho et al., 2013 [31]	Da Vinci Skills Simulator	AI in Simulators				The exercises included needle control and suturing	At Time Point 2, Group 1 showed both increased VR index scores (19.3 ± 4.5 vs. 9.7 ± 4.1; *P* = 0.001) and improved Da Vinci skills, evidenced by higher DV index scores (5.80 ± 1.13 vs. 4.05 ± 1.03; *P* = 0.028) and faster suturing times (7.1 ± 1.54 min vs. 10.55 ± 1.93 min; *P* = 0.018) compared to Group 2	While VR simulator training significantly enhances Da Vinci surgical performance, highly controlled practice conditions may induce an early plateau in the learning curve
Rajesh Kumar et al., 2012 [32]	Da Vinci Skills Simulator	AI in Simulators			Periodic benchmarking executions of 4 minimally invasive surgical skills: Manipulation, Suturing, Transection, and Dissection		Analysis of variance revealed significant differences in mean values at all three points: 1 month (*F* = 71.88 > 2.23), 3 months (*F* = 51.02 > 2.37), and 5 months (*F* = 71.4 > 2.57), all exceeding critical values at α = 0.05	The results demonstrated that trainees’ skill parameters progressively converged toward expert-derived benchmarks over the course of the training protocol
Rajesh Kumar et al., 2011 [33]	Da Vinci Skills Simulator	AI in Simulators			Exercise four basic surgical skills: suturing, manipulation, transaction, and dissection		The classification achieved very high accuracy (91.7% for masters, 88.2% for camera motion) and strong sensitivity	This longitudinal study tracked RAS trainees to proficiency using objective assessment methods for operational and technical skills, implemented across participating institutions to evaluate trainee progress
Ruslan Korets et al., 2011 [34]	Mimic dV-Trainer and da Vinci Surgical System	AI in Simulators			Exercises require endowrist manipulation (EM), camera movement and clutching (CC), needle control (NC), and knot-tying (KT)		No significant baseline differences were observed among the three groups. While Group 3 showed no improvement in the EM/CC domain (*P* = 0.15), Groups 1 and 2 demonstrated significant gains (*P* = 0.039 and *P* = 0.007, respectively)	Curriculum-based training on either the MdVT or dVSS simulator significantly enhanced RAS aptitude, with similar gains observed across the exercise domains shared by both platforms
M. Mukherjee et al., 2009 [35]	Da Vinci Skills Simulator	AI in Simulators		Training consisted of four blocks of virtual surgical tasks, followed by a suturing task on a “life-like” suture pad for testing			The experimental group showed significantly greater pre-to-post-improvements than the control group in both time to completion (*p* < 0.05) and dominant instrument tip distance (*p* < 0.01)	Virtual reality surgical skills training produces significant learning gains that transfer to real robot-assisted laparoscopic procedures
Patrick A. Kenney et al., 2009 [36]	Da Vinci Skills Simulator	AI in Simulators		Each subject completed two EndoWrist modules and two needle-driving modules			Experienced robotic surgeons significantly outperformed novices across nearly all metrics, including total score, task time, total motion, and instrument collisions (*P* < 0.01)	The dV-Trainer demonstrated face, content, and construct validity as a virtual reality simulator for the da Vinci Surgical System
Timothy N. Judkins et al., 2008 [37]	Da Vinci Skills Simulator	AI in Simulators		Throughout the study, subjects used the dVSS to perform and practice three tasks: bimanual carrying (BC), needle passing (NP), and suture tying (ST)			At baseline, experts outperformed novices across all metrics: shorter task completion time (TTC), less total distance traveled (D), lower curvature (j), and higher speed (S) (*p* < 0.05)	This study showed that objective kinematic measures can differentiate between novice and expert performance and detect training effects in robotic surgical tasks
Indran Balasundaram et al., 2008 [38]	Da Vinci Skills Simulator	AI in Simulators		Five tasks of increasing difficulty were selected: needle manipulation, suture without traction, suture with traction, abstract square knot, and interrupted suturing			Significant learning curves were observed across all five tasks. Error scores improved significantly with repetition in only one task. Experts completed two tasks significantly faster than novices	Practice sessions on a VR simulator significantly improve technical performance in robotic surgical tasks
Westwood, J. D., 2007 [39]	Da Vinci Skills Simulator	AI in Simulators		5 trials for each of two tasks: bimanual carrying, (BC); needle passing (NP)			While robot tip speed and elbow range of motion showed no significant differences between environments for either the BC or NP task, time to task completion, and distance traveled differed significantly for both tasks	The VR environment demonstrated validity as a training tool for robotic laparoscopy
Charles Y. RO et al., 2005 [40]	Da Vinci Skills Simulator	AI in Simulators		Seven drills were created to mimic clinical robotic surgical tasks			Across all seven training drills, expert surgeons consistently achieved higher performance scores than novices (*p* < 0.05). The RLC for novices reflected an improvement in scores (*p*<0.05)	This newly developed drill set provides a structured platform for assessing performance in RAS
Ilana Nisky et al., 2013 [41]	Da Vinci Skills Simulator and freehand	A linear mixed model		Participants performed two types of movements: reach and reversal			All users moved slower during teleoperation compared to freehand movements (F19343 = 345; *p*<0.001)	Significant differences between novices and experienced surgeons were observed even in simple point-to-point movements
Amanjot S. Sethi et al., 2009 [42]	Mimic dV-Trainer	AI in Simulators		All participants performed three repetitions of three tasks: Ring and Cone, String Walk, and Letterboard			All 20 participants rated the simulator as average to easy-to-use and above average to high on all realism parameters	The MdVT exhibited excellent face and content validity with reasonable workload, confirming its appropriateness for robotic surgical training
Stéfanie A. Seixas-Mikelus et al., 2010 [43]	The Robotic Surgical Simulator	AI in Simulators		Participants were invited to experience RoSS			79% of participants agreed that RoSS could serve as a tool for privileging or certification in RAS	Based on expert evaluation, the RoSS demonstrated appropriate content validity as a teaching modality for RAS
Andrew J. Hung et al., 2011 [44]	Da Vinci Skills Simulator	AI in Simulators		Trainees underwent a 10-week simulator curriculum			Overall baseline simulator performance correlated strongly with both baseline and final tissue performance (*r* = 0.7, *p* < 0.0001 for both concurrent and predictive validity)	This study documented the concurrent and predictive validity of the Skills Simulator, with the greatest training benefits observed in trainees who entered with low baseline robotic proficiency
Michael Connolly et al., 2013 [45]	Da Vinci Skills Simulator	AI in Simulators	The curriculum included five Da Vinci Skills Simulator tasks: Pick and Place, Camera Targeting 2, Peg Board 2, Matchboard 2, and Suture Sponge 3				Both the mean overall scores and the mean completion times (seconds) significantly differed across groups for every task analyzed	The curriculum represents a valid method for assessing and discriminating between different levels of robotic surgical skill on the Da Vinci Si Surgical System
John Rhodes Martin et al., 2020 [46]	RobotiX Mentor	AI in Simulators	The avian tissue model comprises five technical tasks				Paired t tests revealed significant improvements across all GEARS domains (*p* < 0.001 to *p* = 0.01), as well as in time (*p* < 0.001) and errors (*p* = 0.003) measured by psychometric checklist	Completion of FRS curriculum on the RobotiX Mentor led to significant improvement in robotic surgical skills among novices, demonstrating its effectiveness as a training tool
Ute Walliczek et al., 2015 [47]	Da Vinci Skills Simulator	AI in Simulators	Five exercises were administered in a fixed sequence: Match Board (MB)1, MB2, MB3, then Ring and Rail (RR)1 and RR2				In the final assessment of the novel task (NT), no statistically significant differences were observed between group 1 and group 2 on nearly all measured parameters	Longer intervals between training sessions are assumed to play a secondary role, whereas total repetition frequency appears crucial for improving technical performance
Tarek Alzahrani, et al., 2013 [48]	Da Vinci Skills Simulator	AI in Simulators	Each participant performed nine tasks: Peg Board 2, Match Board 2, Needle Targeting, Ring and Rail 2, Dots and Needles 1, Suture Sponge 2, Energy Dissection 1, Ring Walk 3, and Tubes				For construct validity, experts outperformed novices on all nine tasks (*p* < 0.05), while intermediates outperformed novices on seven of the nine tasks (*p* < 0.05)	The dVSSS demonstrated face, content, and construct validity across urology, gynecology, and general surgery
Timothy N. Judkins et al., 2009 [49]	Da Vinci Skills Simulator	AI in Simulators	Subjects were assigned to five feedback groups (speed, relative phase, grip force, video, and control). EMG data in both time and frequency domains were collected pre- and post-training.				For the NP tasks, the mean EMG amplitude was significantly lower in the POST trial compared to the PRE trial for all recorded muscles (right FCR: *p* = 0.001; left FCR: *p* = 0.023; right ED: *p *< 0.0005; left ED: *p* = (0.001)	Using EMG analysis, clinical biomechanics can elucidate how robotic surgical training impacts motor performance

*RAS* robot-assisted surgery, *dVSS* Da Vinci Skills Simulator, *LS* least-square, *FNN* feedback forward neural networks, *kNN* k-nearest neighbors, *LDA* linear discriminant analysis, *GNB* Gaussian Naive Bayes, *QDA* quadratic discriminant analysis, *LR* logistic regression, *RF* Random Forest, *SVM* support vector machine, *NB* Naive Bayes

### AI in simulators and experimental tasks

A review of the selected studies reveals that AI has been extensively applied across various simulators and experimental tasks in robotic surgical skills training. Among AI-based surgical simulators, the Da Vinci Skills Simulator (dVSS) is the most widely used. While studies investigating the dVSS have utilized a variety of experimental tasks, they provide convergent evidence for its effectiveness in robotic surgical skills training. Wang et al. [14] demonstrated that AI‑enhanced training on the dVSS significantly improved trainees’ basic robotic surgical skills while reducing their cognitive workload. Meier et al. [24] confirmed the dVSS as a practical training tool for all individuals learning or practicing RAS. Patel et al. [26] highlighted that virtual reality robotic simulators such as the dVSS are accessible to general surgeons for skill enhancement. Cho et al. [31] reported that virtual reality simulation training can elevate proficiency in operating the dVSS, noting that controlled VR practice allows the learning curve to plateau at an earlier stage. In addition to the dVSS, LAP MentorTM laparoscopic simulator, Mimic dV-Trainer (MdVT), RobotiX Mentor, and other simulators have also been proven to be effective in training. Kang et al. [25] found that a training regimen of one-hour daily practice sessions over four consecutive days in Mimic dV-Trainer yielded the optimal outcome. This schedule not only produced the highest final scores and demonstrated continuous improvement but also proved to be an effective approach that minimized trainee fatigue. Whittaker et al. [27] assessed the feasibility and acceptability of the construct, face, and content validity of the RobotiX Mentor and served the RobotiX Mentor as an effective tool for both training in and objective assessment of fundamental robotic surgical skills.

For the tasks selected for the experiment, Suturing Task and Needle Passing were the most common, occurring 21 times [14, 17, 20–23, 25, 27, 29, 31–33, 35–38, 40, 45, 46, 48] and 9 times, respectively [15, 18, 19, 29, 31, 37, 39, 47, 49]. Other tasks assessed were 12 [14, 21, 22, 24, 26–28, 32, 34, 40, 45, 48], including Camera Targeting, Ring Walk, Energy Switching, and Energy Dissection. All these tasks are part of the robotic surgical skills training and have achieved excellent training results.

### AI‑based feedback

AI‑based feedback has also been shown to enhance the effectiveness of robotic surgical skills training. Ma et al. [17] demonstrated that AI‑driven feedback improves needle-handling and needle-driving scores, indicating its utility in helping surgical trainees who with initially lower performance acquire robotic operative skills. Vallabhajosula et al. [29] conducted training across three tasks of varying complexity: a simple bimanual carrying (BC) task, an intermediate needle‑passing (NP) task, and a complex suture‑tying (ST) task. Their findings revealed that task‑specific augmented feedback contributes to improved training outcomes. Judkins et al. [49] utilized the da Vinci surgical system to investigate the effects of real-time augmented visual feedback on muscle activation and fatigue during short-term training and concluded that real-time augmented visual feedback can improve training outcomes. Together, these studies substantiate that AI‑based feedback is an effective component of robotic surgical skill acquisition.

### AI‑based training approaches

The studies encompass various AI‑based training approaches, including traditional AI method, machine learning, and deep learning. Tang et al. [15] applied least‑square (LS) regression and feedforward neural networks (FNNs) to predict performance in needle insertion simulators. Their results showed that the FNN achieved over 90% accuracy, outperforming the LS model, which attained a mean accuracy of 78.02% across the defined evaluation metrics. Munawar et al. [20] developed a dynamic simulation environment using machine learning algorithms that enabled real‑time control and collision detection, imparts realistic behavior to needles and sutures, and generates multimodal annotated data, such as labeled depth information. This platform not only supports simulation‑based surgical training but also provides a foundation for machine learning research in surgical scene perception and autonomous action.

These studies also analyzed and compared machine learning-based models for robotic surgical skills training. In their evaluation of machine learning models for performance classification, Prevezanou et al. [16] assessed seven algorithms: Linear Discriminant Analysis (LDA), Quadratic Discriminant Analysis (QDA), Random Forest (RF), the k-Nearest Neighbor (kNN), Support Vector Machine (SVM), Gaussian Naive Bayes (GNB), and Logistic Regression (LR). When each task was analyzed individually, the differences in performance among the algorithms were modest, but certain algorithms exhibited superior performance. Specifically, for Task 5 (Clipping and Grasping), the SVM (Linear) achieved the highest accuracy (97.1%), while LDA yielded the lowest accuracy (89.2%). Moreover, QDA attained the highest sensitivity (98.1%), while LDA demonstrated the best specificity (99.2%). For Task 6 (Two-Handed Maneuvers), SVM (Linear) again showed the highest accuracy (96.9%), while GNB produced the lowest accuracy (92.5%). SVM (Linear) also exhibited the greatest specificity (98.9%), while RF and QDA achieved the highest sensitivity (95.3%). For Task 7 (Cutting), all models showed lower performance scores. LDA obtained the highest accuracy (75.7%), while LR performed the worst (63.6%). Regarding specificity, LDA outperformed the other algorithms (87.8%), whereas QDA reached the highest sensitivity (81.1%). Ershad et al. [23] trained and validated a support vector machine classifier via tenfold cross-validation using sparse codes derived from the training set. Their methods demonstrated a mean improvement in classification accuracy of 18.7 to 68.5% compared to raw data, and 1.35% to 20.59% compared to PCA-based features. Collectively, these studies indicate that integrating machine learning methods into surgical simulators can facilitate automated feedback and performance assessment, thereby enhancing training efficacy.

### AI-based assessment

AI is transforming the assessment of robotic surgical skills by enabling more objective and standardized performance evaluation. Judkins et al. [37] investigated objective metrics of robotic surgical performance by comparing novice and expert surgeons across three training tasks. The authors evaluated five kinematic parameters: task completion time, total distance traveled, movement speed, curvature, and relative phase. Similarly, Kumar et al. [32] developed an AI-driven assessment framework based on Support Vector Machine (SVM) algorithms. The system utilized automatically recorded data from four fundamental robotic surgical skills—suturing, manipulation, cutting, and dissection—to differentiate between expert surgeons and trainees. Using data from six trainees and two expert surgeons, the model achieved a high classification performance, with accuracies of 91.7% and 88.2% for master and camera motion data, respectively, along with high sensitivity. In this study, the AI model used objective motion data to classify skill level, with expert performance serving as the reference standard. Collectively, these studies demonstrate how AI can support objective skill assessment by converting complex performance data into standardized and reproducible metrics, thereby improving the consistency, reliability, and scalability of robotic surgical training evaluations.

## Discussion

This systematic review aims to assess status of the application of AI in robotic surgical skills training in the modern robotic era and future horizons. This systematic review summarizes 36 articles regarding the application of AI in robotic surgical skills training. While AI has developed rapidly and been widely applied, existing research has predominantly explored its use in RAS and laparoscopic surgery procedures. This review specifically focuses on the application of AI in robotic surgical skills training. This review examines AI training simulators, AI-based feedback, and AI training methodologies and models, highlighting their critical importance for advancing robotic surgical education.

The reviewed studies employed a range of AI-based simulators, all studies consistently demonstrated that AI simulation effectively improves robotic surgical performance. Among the available simulators, the most widely used is the Da Vinci Skills Simulator (dVSS), which integrates virtual reality training with the physical da Vinci surgeon console to enhance realism [23]. A key advantage of this system is its ability to capture automated performance metrics (APMs), such as camera motion and instrument control. Machine learning (ML) algorithms analyze this data to identify performance patterns, enabling users to receive objective feedback. For instance, when evaluating an individual’s procedure duration, ML can assess the time taken for each critical stage, allowing for targeted feedback on specific skills. This comprehensive analysis helps users understand both their strengths and areas for improvement.

This platform exhibits excellent face validity and has demonstrated evidence of construct and content validity. However, despite being a high-fidelity training tool, its operational dependency on an actual Da Vinci console limits its accessibility for many institutions. Other robotic surgery training platforms, including the Mimic dV-Trainer (MdVT) and the RobotiX Mentor, are less widely adopted but have demonstrated face, construct, and content validity, supporting their effectiveness as tools for robotic surgical skills training. Nevertheless, each simulator has its own unique characteristics, such as specific training methodologies, task variety, and assessment criteria [27]. This makes it difficult to compare AI-based models derived from data obtained using different simulators. As time progresses, new simulators will continue to emerge, while existing platforms will undergo continuous refinement and development. Consequently, future development efforts remain directed toward creating more accessible and comprehensive simulators, alongside the pursuit of standardized training protocols.

Various machine learning and statistical classification techniques, including Linear Discriminant Analysis (LDA), Quadratic Discriminant Analysis (QDA), Random Forest (RF), Support Vector Machines (SVM), and Artificial Neural Networks (ANN), have been incorporated into automated surgical skill assessment frameworks [15, 16, 32]. While methods such as LDA, QDA, and RF are not artificial intelligence systems in themselves, they serve as important algorithmic components within broader AI- and machine learning-based assessment pipelines. These approaches have been used to classify performance levels, identify patterns in surgical metrics, and support objective skill evaluation. Their inclusion provides valuable methodological context and highlights the evolution of computational approaches that have contributed to the development of AI-assisted surgical training and assessment systems.

Variations in task complexity, motion patterns, feature characteristics, and dataset composition may influence the suitability and performance of different machine learning approaches. However, direct comparisons remain challenging because of the heterogeneity in study designs, performance metrics, datasets, and evaluation protocols across the reviewed literature. The studies included in this review employed a variety of training tasks across different simulators, with the suturing task [14, 17, 20–23, 25, 27, 29, 31–33, 35–38, 40, 45, 46, 48] and needle passing [15, 18, 19, 29, 31, 37, 39, 47, 49] being the most frequently investigated. The widespread use of these tasks likely reflects their suitability for surgical skill assessment. Tasks that are overly simple may not adequately discriminate between novice and expert performance, whereas highly complex tasks can increase training time and introduce additional sources of variability that may affect assessment accuracy. In contrast, suturing and needle passing represent intermediate-complexity tasks that evaluate fundamental technical skills while closely approximating real-world robotic-assisted surgical procedures. Several studies reporting high classification accuracy utilized data derived from suturing and needle passing tasks. However, given the substantial methodological heterogeneity among studies, it is not possible to conclude definitively that these tasks are inherently superior for machine learning model development. Rather, the available evidence suggests that their frequent adoption, clinical relevance, and ability to capture meaningful variations in technical performance make them valuable platforms for developing and evaluating AI-assisted surgical skill assessment systems. Future studies employing standardized datasets and benchmarking protocols are needed to determine whether specific algorithms perform differently across distinct surgical tasks.

A key finding of this review is the growing role of AI in improving the objectivity, standardization, and scalability of robotic surgical skills training and assessment. Traditional assessment approaches frequently rely on expert observation and subjective rating scales, which can be influenced by inter-rater variability, evaluator bias, and resource constraints [50]. As robotic surgery training continues to expand, these limitations highlight the need for more consistent and scalable assessment methods. AI offers a promising solution by enabling the automated analysis of large volumes of performance data, including instrument kinematics, motion trajectories, task completion metrics, historical performance records, and video recordings. Several studies included in this review demonstrate the potential of AI-driven assessment systems to objectively evaluate surgical proficiency. For example, Judkins et al. [37] showed that quantitative kinematic parameters, such as task completion time, total distance travel, movement speed, curvature, and relative phase, could effectively distinguish novice surgeons from experts and detect improvements associated with training. Similarly, Kumar et al. [32] demonstrated that machine learning algorithms could accurately classify surgeon skill level using automatically recorded motion data from fundamental robotic surgical tasks. These findings suggest that AI can identify subtle performance characteristics that may not be consistently recognized through conventional human assessment alone. Beyond improving assessment accuracy, AI-based evaluation systems have the potential to provide continuous real-time feedback and support competency-based training frameworks. By generating standardized and reproducible performance metrics, AI may reduce dependence on expert evaluators and facilitate more efficient monitoring of trainee progression. Such capabilities are particularly valuable in robotic surgery, where access to experienced instructors and opportunities for direct observation may be limited.

AI-based feedback represents a highly promising application of AI in robotic surgical skills training. While initial studies, such as those by Ma et al. [17] and Vallabhajosula et al. [29], have successfully developed explainable AI models that provide basic performance scores or skill level classifications, the feedback remains relatively simplistic. To achieve truly personalized training, future systems must deliver more granular, informative, and actionable feedback. Furthermore, existing review studies have predominantly examined feedback provided after training [17, 29], with limited investigation into determining the ideal timing for its delivery. In future, a comparative evaluation of the three feedback modalities—providing feedback before training, providing feedback during training, and providing feedback after training—is warranted to determine which approach optimizes training effectiveness.

Among the studies included in this systematic review, AI was primarily applied to simulators during the early phase (2000–2020). With the continuous advancement of both AI and the field of robotic surgical skills training, research conducted in recent years (2020–2025) has gradually shifted toward applying AI to automated feedback and assessment, accompanied by an increased use of machine learning methods in this domain. This indicates that AI is increasingly applied in robotic surgical skills training. Although the studies included in this review predominantly employed traditional machine learning approaches, recent advances in deep learning have demonstrated enhanced capabilities for automatically extracting complex features from high-dimensional temporal surgical data, potentially improving skill classification, performance assessment, and motion pattern recognition. In addition, emerging generative AI models offer opportunities for synthetic data generation, data augmentation, and simulation enhancement, which may help overcome current challenges related to limited dataset size, diversity, and generalizability. Together, these developments highlight promising avenues for the development of more robust, scalable, and adaptive AI-driven surgical training systems. Future research using standardized datasets, reporting frameworks, and benchmarking protocols will be essential to evaluate the comparative performance of different AI algorithms across diverse robotic surgical tasks and training environments [51].

This review is subject to several limitations. First, the use of highly specific search terms may have limited the retrieval of potentially relevant studies though our search strategy was designed to maximize specificity and ensure that the included studies directly addressed robot-assisted surgical skill assessment. Second, many of the included studies had small sample sizes and exhibited significant heterogeneity in the simulators, experimental tasks, and assessment methods employed. This diversity precluded a formal meta-analysis to quantitatively synthesize the results. Moreover, a reliance on limited metrics in some studies hindered a comprehensive evaluation of overall robotic surgical performance, such as time, repetition counts, and final scores. Furthermore, in comparative experiments, participants are often categorized as “novices” or “experts,” yet the criteria for expertise, such as case volume or years of experience, are inconsistently defined across studies. These proxies may not reflect actual surgical proficiency. The heterogeneity arising from differences in simulator platforms (e.g., dVSS, Mimic, RobotiX), study timeframes (2005–2025), and data acquisition configurations may have influenced the comparability of reported results and the generalizability of conclusions. In particular, variations in sampling rates, feature extraction methods, and performance metrics limit direct cross-study comparisons and may introduce bias toward frequently used simulators or tasks. Finally, it should be noted that the evidence base is predominantly focused on the Da Vinci platform, with fewer studies investigating other simulators. The lack of direct comparative studies between different simulator platforms further limits the generalizability of the conclusions to the broader field of robotic surgical skills training.

## Conclusion

AI has advanced rapidly and shows considerable potential to enhance trainees’ technical proficiency in robotic surgery, with AI-driven feedback demonstrating significant value in facilitating skill acquisition. Despite these promising developments, the integration of AI into robotic surgical skills training remains in its early stages. The lack of standardized implementation and the limited integration of AI tools into formal robotic surgical skills training continue to hinder broader adoption. Moving forward, the systematic incorporation of AI into training programs—particularly through real-time, personalized feedback grounded in objective performance metrics—could improve training efficiency, accelerate skill acquisition, support competency-based progression, and ultimately enhance both patient safety and surgical outcomes.

## Supplementary Information

Below is the link to the electronic supplementary material.

Supplementary file1 (DOCX 16 KB)

## Data Availability

The data of this systematic review can be obtained from the corresponding author.

## References

[1] Morrell ALG, Morrell-Junior AC, Morrell AG, Mendes JMF, Tustumi F, DE-Oliveira-E-Silva LG, Morrell A (2021) The history of robotic surgery and its evolution: when illusion becomes reality. Rev Col Bras Cir 48:e20202798 10.1590/0100-6991e-20202798 PMC10683436 33470371

[2] Douissard J, Hagen ME, Morel P (2019) The da Vinci Surgical System. In: Domene C, Kim K, Vilallonga Puy R, Volpe P (eds) Bariatric robotic surgery. Springer, Cham

[3] Diana M, Marescaux J (2015) Robotic surgery. Br J Surg 102(2):e15–e28. 10.1002/bjs.9711 25627128

[4] Reddy K, Gharde P, Tayade H, Patil M, Reddy LS, Surya D, Srivani Reddy L (2023) Advancements in robotic surgery: a comprehensive overview of current utilizations and upcoming frontiers. Cureus 15(12) 10.7759/cureus.50415 PMC10784205 38222213

[5] Lanfranco AR, Castellanos AE, Desai JP, Meyers WC (2004) Robotic surgery: a current perspective. Ann Surg 239(1):14–21 10.1097/01.sla.0000103020.19595.7d PMC1356187 14685095

[6] Bickford M, Alruwaili F, Ragab S, Rothenberg H, Abedin-Nasab M (2025) Impact of extended reality on robot-assisted surgery training: a systematic review and meta-analysis. J Robot Surg 19(1):412 10.1007/s11701-025-02559-z PMC12283816 40694147

[7] Rassweiler J, Sentker L, Seemann O, Hatzinger M, Rumpelt HJ (2001) Laparoscopic radical prostatectomy with the Heilbronn technique: an analysis of the first 180 cases. J Urol 166(6):2101–2108 11696715

[8] Narazaki K, Oleynikov D, Stergiou N (2006) Robotic surgery training and performance: identifying objective variables for quantifying the extent of proficiency. Surg Endosc 20(1):96–103 10.1007/s00464-005-3011-3 16374675

[9] Carpenter BT, Sundaram CP (2017) Training the next generation of surgeons in robotic surgery. Robot Surg Res Rev. 10.2147/RSRR.S70552 PMC6193443 30697562

[10] Walliczek-Dworschak U, Mandapathil M, Förtsch A, Teymoortash A, Dworschak P, Werner JA, Güldner C (2017) Structured training on the da Vinci skills simulator leads to improvement in technical performance of robotic novices. Clin Otolaryngol 42(1):71–80 10.1111/coa.12666 27133186

[11] Tergas AI, Sheth SB, Green IC, Giuntoli RL, Winder AD, Fader AN (2013) A pilot study of surgical training using a virtual robotic surgery simulator. JSLS J Soc Laparoendosc Surg 17(2):219 10.4293/108680813X13654754535872 PMC3771788 23925015

[12] Knudsen JE, Ghaffar U, Ma R, Hung AJ (2024) Clinical applications of artificial intelligence in robotic surgery. J Robot Surg 18(1):102 10.1007/s11701-024-01867-0 PMC10907451 38427094

[13] Page MJ, McKenzie JE, Bossuyt PM, Boutron I, Hoffmann TC, Mulrow C D, Moher D (2021) The PRISMA 2020 statement: an updated guideline for reporting systematic reviews. BMJ 372. 10.1136/bmj.n71 PMC8005924 33782057

[14] Wang R, Zheng N, Liang Y, Cui H, Ren T, Xing W, Li Y (2025) Multi-perspective analysis of daVinci surgical virtual reality training: a prospective randomized controlled study. J Robot Surg 19(1):221 10.1007/s11701-025-02309-1 PMC12084236 40377751

[15] Tang R, Yao S, Bai L, Yan H, Meng MQH, Ren H (2024) Data-driven 3-D tactile cues with intermediate soft interfaces toward training needle insertions. IEEE Sens J 24(5):7205–7213

[16] Prevezanou K, Seimenis I, Karaiskos P, Pikoulis E, Lykoudis PM, Loukas C (2024) Machine learning approaches for evaluating the progress of surgical training on a virtual reality simulator. Appl Sci 14(21):9677

[17] Ma R, Kiyasseh D, Laca JA, Kocielnik R, Wong EY, Chu TN, Cen S, Yang CH, Dalieh IS, Haque TF, Goldenberg MG, Huang X, Anandkumar A, Hung AJ (2024) Artificial intelligence-based video feedback to improve novice performance on robotic suturing skills: a pilot study. J Endourol 38(8):884–891 10.1089/end.2023.0328 PMC11947633 37905524

[18] Turbati MS, Goldblatt MI, Gould JC, Higgins RM (2023) Robotic simulation: validation and qualitative assessment of a general surgery resident training curriculum. Surg Endosc 37(3):2304–2315 10.1007/s00464-022-09558-3 36002680

[19] Brown JD, Kuchenbecker KJ (2023) Effects of automated skill assessment on robotic surgery training. Int J Med Robot Comput Assist Surg 19(2):e2492 10.1002/rcs.2492 36524325

[20] Munawar A, Wu JY, Fischer GS, Taylor RH, Kazanzides P (2022) Open simulation environment for learning and practice of robot-assisted surgical suturing. IEEE Robot Autom Lett 7(2):3843–3850

[21] Wu C, Cha J, Sulek J, Sundaram CP, Wachs J, Proctor RW, Yu D (2021) Sensor-based indicators of performance changes between sessions during robotic surgery training. Appl Ergon 90:103251 10.1016/j.apergo.2020.103251 PMC7606790 32961465

[22] Wu C, Cha J, Sulek J, Zhou T, Sundaram CP, Wachs J, Yu D (2020) Eye-tracking metrics predict perceived workload in robotic surgical skills training. Hum Factors 62(8):1365–1386 10.1177/0018720819874544 PMC7672675 31560573

[23] Ershad M, Rege R, Majewicz Fey A (2019) Automatic and near real-time stylistic behavior assessment in robotic surgery. Int J Comput Assist Radiol Surg 14(4):635–643 10.1007/s11548-019-01920-6 30779023

[24] Meier M, Horton K, John H (2016) Da Vinci© skills simulator™: is an early selection of talented console surgeons possible? J Robot Surg 10(4):289–296 10.1007/s11701-016-0616-6 27334771

[25] Kang SG, Ryu BJ, Yang KS, Ko YH, Cho S, Kang SH, Patel VR, Cheon J (2015) An effective repetitive training schedule to achieve skill proficiency using a novel robotic virtual reality simulator. J Surg Educ 72(3):369–376 10.1016/j.jsurg.2014.06.023 25481802

[26] Patel A, Patel M, Lytle N, Toro JP, Medbery RL, Bluestein S, Perez SD, Sweeney JF, Davis SS, Lin E (2014) Can we become better robot surgeons through simulator practice? Surg Endosc 28(3):847–853 10.1007/s00464-013-3231-x 24122244

[27] Whittaker G, Aydin A, Raison N, Kum F, Challacombe B, Khan MS, Dasgupta P, Ahmed K (2016) Validation of the RobotiX mentor robotic surgery simulator. J Endourol 30(3):338–346 10.1089/end.2015.0620 26576836

[28] Zhang N, Sumer BD (2013) Transoral robotic surgery: simulation-based standardized training. JAMA Otolaryngol Head Neck Surg 139(11):1111–1117 10.1001/jamaoto.2013.4720 24051580

[29] Vallabhajosula S, Judkins TN, Mukherjee M, Suh IH, Oleynikov D, Siu KC (2013) Skills learning in robot-assisted surgery is benefited by task-specific augmented feedback. Surg Innov 20(6):639–647 10.1177/1553350613484590 23575913

[30] Brinkman WM, Luursema JM, Kengen B, Schout BM, Witjes JA, Bekkers RL (2013) Da Vinci skills simulator for assessing learning curve and criterion-based training of robotic basic skills. Urology 81(3):562–566 10.1016/j.urology.2012.10.020 23295136

[31] Cho JS, Hahn KY, Kwak JM, Kim J, Baek SJ, Shin JW, Kim SH (2013) Virtual reality training improves da Vinci performance: a prospective trial. J Laparoendosc Adv Surg Tech A 23(12):992–998 10.1089/lap.2012.0396 24138400

[32] Kumar R, Jog A, Vagvolgyi B, Nguyen H, Hager G, Chen CCG, Yuh D (2012) Objective measures for longitudinal assessment of robotic surgery training. J Thorac Cardiovasc Surg 143(3):528–534 10.1016/j.jtcvs.2011.11.002 PMC3288290 22172215

[33] Kumar R, Jog A, Malpani A, Vagvolgyi B, Yuh D, Nguyen H, Hager G, Chen CCG (2012) Assessing system operation skills in robotic surgery trainees. Int J Med Robot 8(1):118–124 10.1002/rcs.449 PMC3312785 22114003

[34] Korets R, Mues AC, Graversen JA, Gupta M, Benson MC, Cooper KL, Landman J, Badani KK (2011) Validating the use of the Mimic dV-trainer for robotic surgery skill acquisition among urology residents. Urology 78(6):1326–1330 10.1016/j.urology.2011.07.1426 22001096

[35] Mukherjee M, Siu KC, Suh IH, Klutman A, Oleynikov D, Stergiou N (2009) A virtual reality training program for improvement of robotic surgical skills. Medicine meets virtual reality 17. IOS Press, pp 210–214 19377151

[36] Kenney PA, Wszolek MF, Gould JJ, Libertino JA, Moinzadeh A (2009) Face, content, and construct validity of dV-trainer, a novel virtual reality simulator for robotic surgery. Urology 73(6):1288–1292 10.1016/j.urology.2008.12.044 19362352

[37] Judkins TN, Oleynikov D, Stergiou N (2009) Objective evaluation of expert and novice performance during robotic surgical training tasks. Surg Endosc 23(3):590–597 10.1007/s00464-008-9933-9 18443870

[38] Balasundaram I, Aggarwal R, Darzi A (2008) Short-phase training on a virtual reality simulator improves technical performance in tele-robotic surgery. Int J Med Robot 4(2):139–145 10.1002/rcs.181 18327876

[39] Westwood JD (2007) Virtual reality for robotic laparoscopic surgical training. Med Meets Virtual Real 15 Vivo Vitro Silico Desig Next Med 125:127 17377249

[40] Charles Y, Ioannis K, Ashton Robert C Jr (2005) A novel drill set for the enhancement and assessment of robotic surgical performance. Stud. Health Technol. Inform 111:418–421 15718771

[41] Nisky I, Okamura AM, Hsieh MH (2014) Effects of robotic manipulators on movements of novices and surgeons. Surg Endosc 28(7):2145–2158 10.1007/s00464-014-3446-5 PMC8101070 24519031

[42] Sethi AS, Peine WJ, Mohammadi Y, Sundaram CP (2009) Validation of a novel virtual reality robotic simulator. J Endourol 23(3):503–508 10.1089/end.2008.0250 19265469

[43] Seixas‐Mikelus SA, Stegemann AP, Kesavadas T, Srimathveeravalli G, Sathyaseelan G, Chandrasekhar R, Wilding GE, Peabody JO, Guru KA (2011) Content validation of a novel robotic surgical simulator. BJU Int 107(7):1130–1135 10.1111/j.1464-410X.2010.09694.x 21029316

[44] Hung AJ, Patil MB, Zehnder P, Cai J, Ng CK, Aron M, Gill IS, Desai MM (2012) Concurrent and predictive validation of a novel robotic surgery simulator: a prospective, randomized study. J Urol 187(2):630–637 10.1016/j.juro.2011.09.154 22177176

[45] Connolly M, Seligman J, Kastenmeier A, Goldblatt M, Gould JC (2014) Validation of a virtual reality-based robotic surgical skills curriculum. Surg Endosc 28(5):1691–1694 10.1007/s00464-013-3373-x 24380993

[46] Martin JR, Stefanidis D, Dorin RP, Goh AC, Satava RM, Levy JS (2021) Demonstrating the effectiveness of the fundamentals of robotic surgery (FRS) curriculum on the RobotiX Mentor virtual reality simulation platform. J Robot Surg 15(2):187–193 10.1007/s11701-020-01085-4 32409995

[47] Walliczek U, Förtsch A, Dworschak P, Teymoortash A, Mandapathil M, Werner J, Güldner C (2016) Effect of training frequency on the learning curve on the da Vinci skills simulator. Head Neck 38(S1):E1762–E1769 10.1002/hed.24312 26681572

[48] Alzahrani T, Haddad R, Alkhayal A, Delisle J, Drudi L, Gotlieb W, Anidjar M (2013) Validation of the da Vinci Surgical Skill Simulator across three surgical disciplines: A pilot study. Canadian Urolog Assoc J 7(7–8):E520 10.5489/cuaj.419 PMC3713157 23914275

[49] Judkins TN, Oleynikov D, Stergiou N (2009) Electromyographic response is altered during robotic surgical training with augmented feedback. J Biomech 42(1):71–76 10.1016/j.jbiomech.2008.09.039 19041972

[50] Andersen DK (2012) Fundamentals of surgical simulation: principles and practices. JAMA 307(9):974–975

[51] Collins JW, Marcus HJ, Ghazi A, Sridhar A, Hashimoto D, Hager G, Arezzo A, Jannin P, Maier-Hein L, Marz K, Valdastri P, Mori K, Elson D, Giannarou S, Slack M, Hares L, Beaulieu Y, Levy J, Laplante G, Ramadorai A, Jarc A, Andrews B, Garcia P, Neemuchwala H, Andrusaite A, Kimpe T, Hawkes D, Kelly JD, Stoyanov D (2022) Ethical implications of AI in robotic surgical training: a Delphi consensus statement. Eur Urol Focus 8(2):613–622 10.1016/j.euf.2021.04.006 33941503

