# Supplementary material for: Status of the application of artificial intelligence (AI) in robotic surgical skills training in the modern robotic era and future horizons: a systematic review
Source: Surg Endosc. 2026 Sep 28;40(11):9357–77. doi: 10.1007/s00464-026-13396-y (PMC13754765; doi:10.1007/s00464-026-13396-y)
Supplement: Supplementary file 1 [file 464_2026_13396_MOESM1_ESM.docx]

**Supplementary Table. Full search strategies for all databases and registers in accordance with PRISMA 2020**

(The table is structured to report the information recommended for transparent reporting of database searches, including the information source, search period, complete search strategy, date searched, and number of records retrieved.)

| **Database** | **Search period** | **Full search strategy** | **Search date** | **Records retrieved** |
| --- | --- | --- | --- | --- |
| **PubMed/MEDLINE** | 1 January 2000–1 October 2025 | (“machine learning” OR “artificial intelligence” OR “AI” OR “deep learning”) AND (“Da Vinci training” OR “robotic surgical skills training” OR “Da Vinci simulator” OR “robotic surgery training” OR “technical robotic skills assessment” OR “Fundamental Skills of Robotic Surgery” OR “FSRS”) | 6 October 2025 | **2,131** |
| **Ovid Embase** | 1 January 2000–1 October 2025 | **1.** exp Artificial Intelligence/ — 17,689 **2.** (machine learning OR artificial intelligence OR AI OR deep learning): ti,ab,kw — 3,702 **3.** 1 OR 2 — 18,537 **4.** Robotic Surgical Procedures/ — 14,842 **5.** (da Vinci training OR robotic surgical skills training OR da Vinci simulator OR robotic surgery training OR technical robotic skills assessment OR fundamental skills of robotic surgery OR FSRS): ti,ab,kw — 5,166 **6.** 4 OR 5 — 17,034 **7.** 3 AND 6 — **274** | 10 October 2025 | **274** |
| **Scopus** | 1 January 2000–1 October 2025 | TITLE-ABS-KEY (“machine learning” OR “artificial intelligence” OR “AI” OR “deep learning”) AND (“Da Vinci training” OR “robotic surgical skills training” OR “Da Vinci simulator” OR “robotic surgery training” OR “technical robotic skills assessment” OR “Fundamental Skills of Robotic Surgery” OR “FSRS”) | 9 October 2025 | **973** |
| **Web of Science** | 1 January 2000–1 October 2025 | TS= (“machine learning” OR “artificial intelligence” OR “AI” OR “deep learning”) AND TS= (“Da Vinci training” OR “robotic surgical skills training” OR “Da Vinci simulator” OR “robotic surgery training” OR “technical robotic skills assessment” OR “Fundamental Skills of Robotic Surgery” OR “FSRS”) | 8 October 2025 | **811** |
| **Cochrane Library** | 1 January 2000–1 October 2025 | **#1.** MeSH descriptor: [Artificial Intelligence] explode all trees — 4,318 **#2.** (machine learning): ti,ab,kw OR (artificial intelligence): ti,ab,kw OR (AI): ti,ab,kw OR (deep learning): ti,ab,kw — 16,669 **#3.** #1 OR #2 — 18,449 **#4.** MeSH descriptor: [Robotic Surgical Procedures] explode all trees — 1,217 **#5.** (Da Vinci training): ti,ab,kw OR (robotic surgical skills training): ti,ab,kw OR (Da Vinci simulator): ti,ab,kw OR (robotic surgery training): ti,ab,kw OR (technical robotic skills assessment): ti,ab,kw OR (Fundamental Skills of Robotic Surgery): ti,ab,kw OR (FSRS): ti,ab,kw — 500 **#6.** #4 OR #5 — 1,583 **#7.** #3 AND #6 — **211** | 10 October 2025 | **211** |
| **Total** | — | — | — | **4,400** |
